# Evaluation of PROforma as a language for implementing medical guidelines in a practical context

**DOI:** 10.1186/1472-6947-6-20

**Published:** 2006-04-05

**Authors:** David R Sutton, Paul Taylor, Kenneth Earle

**Affiliations:** 1 Oxford Brookes University, Oxford, UK; 2 Centre for Health Informatics and Multiprofessional Education, Royal Free & University College Medical School University College London, UK

## Abstract

**Background:**

PROforma is one of several languages that allow clinical guidelines to be expressed in a computer-interpretable manner. How these languages should be compared, and what requirements they should meet, are questions that are being actively addressed by a community of interested researchers.

**Methods:**

We have developed a system to allow hypertensive patients to be monitored and assessed without visiting their GPs (except in the most urgent cases). Blood pressure measurements are performed at the patients' pharmacies and a web-based system, created using PROforma, makes recommendations for continued monitoring, and/or changes in medication. The recommendations and measurements are transmitted electronically to a practitioner with authority to issue and change prescriptions.

We evaluated the use of PROforma during the knowledge acquisition, analysis, design and implementation of this system. The analysis focuses on the logical adequacy, heuristic power, notational convenience, and explanation support provided by the PROforma language.

**Results:**

PROforma proved adequate as a language for the implementation of the clinical reasoning required by this project. However a lack of notational convenience led us to use UML activity diagrams, rather than PROforma process descriptions, to create the models that were used during the knowledge acquisition and analysis phases of the project. These UML diagrams were translated into PROforma during the implementation of the project.

**Conclusion:**

The experience accumulated during this study highlighted the importance of *structure preserving design*, that is to say that the models used in the design and implementation of a knowledge-based system should be structurally similar to those created during knowledge acquisition and analysis. Ideally the same language should be used for all of these models. This means that great importance has to be attached to the *notational convenience *of these languages, by which we mean the ease with which they can be read, written, and understood by human beings. The importance of notational convenience arises from the fact that a language used during knowledge acquisition and analysis must be intelligible to the potential users of a system, and to the domain experts who provide the knowledge that will be used in its construction.

## Background

We have implemented a distributed system for the management of hypertensive patients. The study is one of a number of initiatives in which the management of chronic conditions is addressed by improving the connections between different community-based professionals and, specifically, extending the traditional role of pharmacists in dispensing medicines to allow them to give advice based on measurements of their patients' conditions. This system is being used in a small study in which patients' blood pressures are monitored by their local pharmacists. Six community pharmacies are participating. Initially 250 suitable patients will be identified by the pharmacists from their prescriptions and invited to participate.

The project builds on earlier work evaluating a stand-alone computer-based decision support tool [1]. In this project we sought to overcome significant practical difficulties of the earlier system by using a distributed approach. The pharmacists have access, via a web-based interface, to a computerised clinical guideline, which advises how the patient should be managed. If the system infers that a patient's medication needs to be altered then this information is passed on to a medical practitioner (GP) with authorisation to prescribe. The system will provide the practitioner with advice as to how the patient's medication should best be modified. The practitioner will contact the patient by phone in order to arrange modification of the patient's prescription. In extreme cases the system may advise that the patient should leave the study and be cared for in a conventional manner. Although a limited history is stored on the system it is associated with a study number so that no identifiable patient data is stored on the system.

The system provides potential benefits to patients and healthcare providers in that it should be cheaper and more convenient for patients to visit their pharmacists rather than their GP's surgery.

In this paper we present an account of the development of the system. Our account focuses on the formalism used to represent the clinical guideline and is intended as a qualitative evaluation of the appropriateness of the guideline representation language, PROforma, in a practical setting.

### Clinical guidelines

Systematic reviews of randomised controlled trials have shown that clinical guidelines are an effective tool for improving the quality of care and changing clinical practice [2]. Healthcare professionals have, however, complained that hypertension guidelines are too complex, that the documentation associated with the guidelines is cumbersome and that there are simply too many guidelines for compliance to be practical [2-6]. The computerisation of such guidelines and their integration into systems that support routine clinical work may be one way in which the demand for increased use of guidelines can be reconciled with the concerns of clinicians. At least one trial has shown that a computerised guideline had a greater impact on care than the same guideline in a paper form [6].

Computerised guidelines are an attractive paradigm for clinical decision support tools, since much of the knowledge contained in guidelines has already been rendered explicit. A number of groups have now developed languages that express medical guidelines and processes, with the hope that non-programmers will be able to create computerised clinical guidelines. These are examples of *knowledge representation languages*. In the next two sections we consider guideline and process representation languages, concentrating on one in particular, PROforma.

### Computer interpretable guideline representation languages

A computer interpretable guideline (CIG) is a representation of the knowledge that is needed in order for a computer system to advise clinicians in a way that adheres to guidelines for clinical practice. A number of knowledge representation languages have been developed specifically for the purpose of representing such knowledge. These languages include Arden Syntax[7,8], Asbru [9,10], EON[11], GLIF [12-14] GUIDE [15,16], PRODIGY [17], and PROforma [18-20].

The Arden syntax is a rule-based formalism that is used to create *Medical Logic Modules *(MLM), each of which encodes the logic necessary for an individual medical decision. A MLM contains information representing the context in which an individual rule may become relevant, the logical conditions necessary for it to be activated, and the action that is performed when it is activated. Asbru, EON, GLIF, GUIDE, PRODIGY, and PROforma are languages that permit the description of "Task Network Models". Such models represent sets of interacting medical decisions and actions that are carried out in sequence or in parallel over a period of time. These six languages have been compared in a recent paper by Peleg et al. [21], who have identified areas of common ground, as well as significant differences, between the languages.

All six languages can be used to describe *plans*, where a plan can be defined, as in the Merriam-Webster dictionary, to be "an orderly arrangement of parts of an overall design or objective". The languages differ slightly as to what they consider the elements of a plan to be, and how its objective is expressed. However all allow plans to contain, among other things, decisions, actions, and nested sub-plans. All contain expression languages that represent criteria which influence decisions and control plan execution (e.g. to express conditions that determine whether a task might be started or terminated).

### PROforma

PROforma is a knowledge representation language that can be used to create descriptions of processes that unfold over time and require the cooperation of various actors, such as clinicians or other medical personnel. The language benefits from an easy-to-use graphical editor, which can be obtained by agreement with the research team developing the language. This, and the fact that the authors had some previous experience with PROforma, led us to choose it as the description language to be evaluated in this study.

The use of Process Descriptions is illustrated in. A Process Description is loaded into a software component referred to as the PROforma Engine which maintains a record of the dynamic state of the process, this includes information on which tasks have been performed, which need (or need not) be performed, and the values of any data items associated with the process. The PROforma engine implements a set of operations (the Engine Interface) which allow other components to read or change the state of the guideline in certain predefined ways. In general the execution of the process will require actions to be performed by external actors (e.g. clinicians) who will interact with the engine via some set of user interfaces.

A Process Description is composed of objects drawn from the classes set out diagrammatically in figure 2. Each class of object has a set of named properties (to avoid clutter, properties are not shown in figure 2). Each instance of a given class will have different values for these properties. If one class of object is connected to another by a "kind of" relationship then the more specific class inherits all the properties of the more general class, for instance a PROforma Component has a property named "Description" and hence Tasks, Data Items and all other kinds of PROforma Component will also have this property.

**Figure 1:**
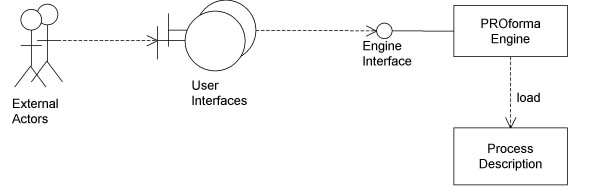
Use of PROforma.

**Figure 2:**
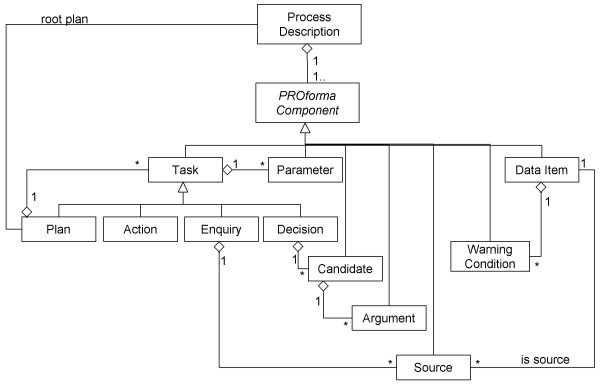
PROforma Classes.

**Figure 3:**
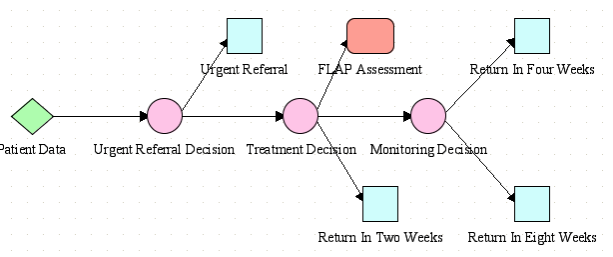
PROforma Process Description.

**Figure 4:**
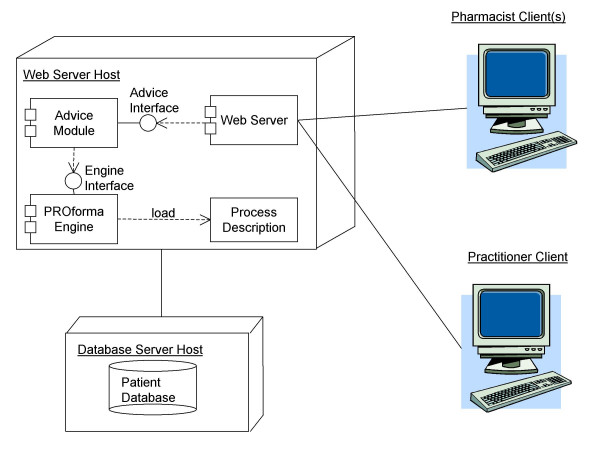
Deployment of the Process Description.

**Figure 5:**
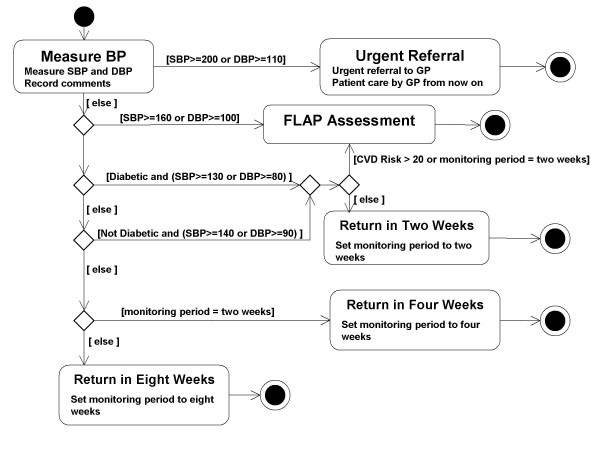
Top Level Plan Expressed as a UML Activity Diagram.

A Process is defined in PROforma as a set of PROforma Components. A PROforma Component can be, among other things, a Task or a Data item. A Task can be an Action, an Enquiry, a Decision or a Plan. An Action generally represents a request for an external actor to do something (e.g. prescribe a drug, or perform some other clinical intervention). An Enquiry represents a request to an external actor to provide values for data items (the Sources of the Enquiry). A Decision represents a choice between one or more Candidates. The choice of Candidate(s) may be performed by an external actor or it may be made automatically by the system. The engine keeps a record of which Candidate(s) have been chosen in which Decisions and this information can be used to control the subsequent execution of the Process. Each Candidate is associated with one or more Arguments, logical expressions which if evaluated as true influence the recommendation of a Candidate.

PROforma includes an expression language that is used to define, among other things, the preconditions that must be true for a task to be activated and the criteria that must be fulfilled for an Argument to be true. The PROforma expression language includes the usual logical, arithmetic, and comparison operators, as well as functions that evaluate the execution states of tasks (i.e. whether they have been, or need to be, performed) as well as the values of data items.

The PROforma language has been given a precise syntax and semantics [18,22]. The semantics defines the value that is returned when an expression is evaluated as well as the way in which the process state changes when Engine Interface operations are invoked. Two implementations of a PROforma engine are available, the Arezzo implementation, which is available commercially from InferMed Ltd. (London, UK) and the Tallis implementation from Cancer Research UK.

PROforma has been used in a variety of research projects and a smaller number of clinical applications. Although publications have arisen from some of these projects, little has been written about the experience of using PROforma in practice. In this paper we present an account of the development of a PROforma guideline for the management of hypertension and evaluate its suitability in a distributed decision aid to be used by pharmacists. First we present a brief discussion of this kind of task, which is to say of knowledge engineering and knowledge representation, in order to identify suitable criteria for the evaluation.

### Knowledge engineering principles

Knowledge engineering involves using computers to perform knowledge-intensive tasks. Implementing a knowledge intensive system requires the co-operation of a number of participants, who can be divided into those who are principally interested in *what *the system does (e.g. domain experts, who supply the knowledge embodied in the system, or potential users, who provide the information about requirements) and those concerned with *how *it does it (e.g. knowledge engineers, who analyse and model the system, or system developers, who develop it).

The development of a knowledge intensive system includes three phases:

• *knowledge acquisition *in which knowledge engineers and domain experts create a shared model of a domain expert's knowledge,

• a process in which a *design *is agreed for a system,

• *system implementation *in which the model is instantiated in the designed software system.

We argue for a "model view" [23] as opposed to a "transfer view" of the knowledge engineering process. That is to say that knowledge engineers and domain experts should construct a shared model of knowledge rather than transferring knowledge expressed by the expert in, for example, natural language into a format intelligible only to the knowledge engineer.

We also argue that the development process should be *structure preserving *[23] the models used in the design and implementation of the system should have the same structure as those developed during knowledge acquisition and analysis. This is because participants interested in what a knowledge-based system does, will often need to talk to participants with an interest in how it does it. Such dialogue occurs during knowledge acquisition; it occurs when the requirements of the system are drawn up; and it occurs when the output of the system needs to be explained or when explanations need to be provided by the system. These dialogues are greatly facilitated if the models that describe what the system does and the models that describe how it does it have a similar structure.

This implies that the models constructed during knowledge acquisition should be reused in the implementation of the system. In order to achieve this it is necessary to create models that are intelligible to domain experts and interpretable by computer systems. The same philosophy underpins, for example, the use of objects as a consistent metaphor across both object-oriented software design and object-oriented programming languages. The process of building a representation of the domain expert's knowledge, therefore, straddles different phases of the software development lifecycle. It involves decisions about the system's functionality. The resulting representation may be simply a documentation of a design, but, depending on the development methodology, a key component of the software may also be programmed in the course of building the model. In this evaluation we assess the adequacy of PROforma and the Tallis implementation as a knowledge engineering tool under three headings: knowledge acquisition, system design and implementation, noting that in practice these three phases overlap.

### Knowledge representation principles

The knowledge in a knowledge-based system must be described and stored in a computable form. Knowledge representation languages are computer languages that facilitate such descriptions. There are various criteria by which such languages can be assessed [24]: *logical adequacy *can the language to represent all the distinctions that one might want to make? *heuristic power *does the language allow the system to draw the required inferences and solve problems within its intended domains of application? *notational convenience *can the language can be read, written, and understood (by humans as well as by computer systems)? *explanatory support *does the language make it easy for a knowledge based system to explain to a user the chain of reasoning that led it to perform a particular action or to reach a particular conclusion?

## Methods

In this section we briefly describe the development of the software and the evaluation of PROforma. The project can be viewed as having three, somewhat overlapping, phases: knowledge acquisition, system design, and implementation. The suitability of PROforma as a guideline representation formalism for this application will have implications at each stage. In this section we present a short account of each stage of the project, indicating the rationale for the different decisions that were made.

### Knowledge acquisition

The guideline in the computerised system was based on that disseminated by the British Hypertension Society [25]. A customised version of the guideline was produced, in a paper form, by the lead clinician on the project. This was then discussed with the other members of the team, both of whom had experience in the development of computerised guidelines, and in a wider forum, including input from a clinical pharmacist. These discussions identified a number of ambiguities which required further clinical input. As explained in later sections, these discussions required the use of paper diagrams which we found were most conveniently created as UML Activity Diagrams, an example of which is presented in figure 5. The modelled activities are presented as rounded rectangles, or activity states. The text enclosed in square brackets associated with arrows indicates the conditions that must be fulfilled for the transitions between states.

### System design

The concept of a web-based tool emerged from our experience with an earlier, stand- alone, system, an attempt to provide a stand alone decision tool. The design was refined through a series of meetings in which the first author played the role of a systems analyst with the third author acting as a customer. Use Case diagrams were used to support the dialogue and help establish the precise requirements of the system.

The specification of the design has implications for the representation of the clinical guideline. The intended users of the system are pharmacists and GPs. The pharmacists must decide how frequently to monitor patients, when to refer to them to GPs, and under what circumstances such a referral becomes a matter of urgency. The reasoning required for these decisions is essentially that needed to set target blood pressures and to identify when deviations from these target pressures become significant and/or a matter of urgent concern. The advice provided to GPs in our system is somewhat less detailed than that provided by other hypertension systems that have appeared in the literature (e.g. those constructed for the comparative study by Peleg et al.) as some decisions are left to the discretion of the GPs themselves. For instance, if a patient is to be prescribed more than one anti-hypertensive agent, then the choice of the second agent is left to the GP. The reason for this division of responsibilities is that we felt that it would be easier to gain the confidence of the various stakeholders in the system if the more detailed decisions were delegated to GPs, even though it would in principle be possible to incorporate them in the process description.

### System implementation

The guideline was modelled using the Tallis implementation of PROforma. The Web interface was built specifically for this study using Java Servlets/JSP and consists of pages containing nothing other than simple HTML in order to avoid making assumptions about the capabilities of the browsers used by the pharmacists. In order to support this project's requirement for an enduring record of the patient's interaction with the system, an SQL database was designed and implemented and an interface created between this and the Tallis PROforma Engine.

Figure 4 illustrates how the process description is deployed and used in the system. The process description is loaded into a PROforma engine. An *advice module *interrogates the state of the Process Description to provide advice to the pharmacist and patient, who access the system over the Web. The advice module provides an abstraction of the PROforma process description, exposing certain details and concealing others. The advice module makes certain assumptions about the state of the guideline, for instance it assumes that only one action will be active at any given time.

PROforma process descriptions can be described graphically using a convention in which squares represent Actions, circles represent Decisions, lozenges represent Enquiries, and round-edged rectangles represent Plans. Using these conventions the root (top-level) plan of the Process definition used in our study can be set out as in figure 3. The Enquiry *Patient Data *represents the step in the process at which data about patient state are acquired from external actors. The three Decisions *Urgent Referral Decision*, *Treatment Decision *and *Monitoring Decision *are points at which choices are made between various candidates. The logic underlying these choices is represented by Candidates and Arguments that are associated with the decisions, but which are not displayed graphically. The choice of Candidates will determine which Actions are performed. The four Actions in the root plan represent instructions that the patient be urgently referred for treatment, or return for further monitoring in two, four, or eight weeks time. The sub-plan *FLAP Assessment *is invoked when the logic encoded in the Decisions implies that the patient's medication should be altered. The plan contains further Decisions and Actions not described here.

The Actions in the Process Description have a number of user-defined parameters that express the values of various properties such as whether the action implies a change to the patient's medication, or the amount of time that should elapse until the next visit.

The arrows between the tasks in the figure represent *scheduling constraints*, that prevent one task from being activated until another has finished, for instance, the Action *Urgent Referral *cannot be activated unless the Decision *Urgent Referral Decision *has completed. Scheduling constraints express necessary, but not sufficient, conditions for the activation of tasks: the completion of the *Urgent Referral Decision *does not guarantee that the Action *Urgent Referral *will take place since each task has a set of logical preconditions that are evaluated after its scheduling constraints are satisfied, and the task is only activated if both its scheduling constraints and its preconditions are satisfied.

### Evaluation

In this project PROforma was used in conjunction with other modelling tools (UML) and the engine was integrated with other elements of software. The work therefore provides a richer test of the language than previous comparative studies and allows us to examine the advantages of specialised approaches to guideline modelling, such as PROforma, in the context of a realistic software engineering project.

The evaluation described here is qualitative, and some of our comments are necessarily subjective. The essential data for the evaluation was generated by the first author, in the form of comments based on his experience as the developer. His comments were discussed with other members of the team and a consensus process used to assess the performance of PROforma against each of the identified desirable characteristics of knowledge representation languages (logical adequacy, heuristic power, notational convenience, and explanatory support) at each stage of the project. These were operationalised in terms that allowed a range of responses, identifying specific strengths and weaknesses. These are categorised by reference first to the phases of knowledge acquisition, system design and implementation and then to the four characteristics.

## Results

In this section we present summary conclusions of our assessment of PROforma in each phase of the project. Detailed discussion of the more important points is presented in the next section.

### Knowledge acquisition

#### Logical adequacy

We found that most of the logic of the medical process involved could be readily described using the constructs provided by PROforma. The process, although complex when taken as a whole, can be broken down into individual decisions that can be represented using simple arithmetic comparisons and propositional logic. However, we noted the following weaknesses:

• limited support for constraints

The actions described in the process description used in our system are intended to be mutually exclusive (if one action is performed then the others are not), and the advice module described in assumes that this constraint will be obeyed. However PROforma provides no mechanism by which such a constraint can be expressed, checked, or enforced.

• limited support for structured data

The value of a data item used in a PROforma process may be atomic (e.g. an integer or string) or it may be an ordered list of atomic values, all of the same type. However the Tallis implementation of PROforma provides no support for record structures or for collections other than ordered lists (The Arezzo implementation has some facilities for representing complex objects but does not place any constraints on the classes of these objects).

#### Heuristic power

The term heuristic power is used to describe the extent to which a knowledge representation language allows a knowledge-based system to draw inferences and solve problems within its intended domains of application. The PROforma language proved adequate to encode the reasoning required.

#### Notational convenience

Acquiring knowledge from a domain expert involves the construction, validation, and refinement of models. The principle of structure preserving design means that, in an ideal world, these models should be reused during the implementation of the eventual system. If we aim at a structure preserving design then notational convenience, i.e. the ease with which a knowledge representation language can be read, written, and understood, acquires a greater importance than the word "convenience" might suggest. If our knowledge representation language does not allow us to create models that can be understood by a domain expert then we will have to use some other formalism to create such models and therefore risk losing the advantages of structure preserving design. We noted the following weaknesses in the PROforma notation:

• graphical representation

During the knowledge acquisition phase of this project it was frequently necessary to express models on paper. Conventions for expressing models graphically are therefore of great importance. PROforma can be represented as text and also in a graphical form, as illustrated in figure 3. The syntax of the textual representation has been given a complete definition in EBNF [18,22] however the graphical representation is less completely defined. De facto conventions exist for the graphical representation of tasks and the scheduling constraints that link them; however other constructs of the PROforma language have no conventional graphical representation. Because of this we frequently made use of UML activity diagrams in the knowledge acquisition phase, and translated the contents of these into PROforma during system implementation.

• duplication of arguments

It also became apparent during the study that the PROforma notation occasionally requires designers to duplicate elements of a process. As can be seen in figure 2, the relationship between PROforma Candidates and Arguments is one to many. However in practice it often desirable to associate an Argument with more than one Candidate, for instance an Argument in favour of one Candidate may be an Argument against another. In PROforma this can only be expressed by creating multiple copies of Arguments. This is notationally inconvenient and carries the risk that errors may be introduced into a Process Description as it is refined, because a designer may update one copy of an Argument but omit to do the same to other copies.

#### Explanatory support

A knowledge-based system should be capable of explaining to its users the reasons why it has recommended a particular course of action, or drawn a particular inference. We use the term explanatory support to describe the extent to which a knowledge representation language facilitates such explanation.

The PROforma Argument and Candidate constructs offer a natural way to present explanations to a user that relates well to the manner in which such explanations are presented in ordinary human dialogue. The Argument class has *Description *and *Caption *properties, which it inherits from the PROforma Component class. The values of these properties are text descriptions of the Argument, the difference between them lies only in their intended use, the Caption property is intended to provide a short description of a component and the Description a longer one. The value of a Caption or Description can be expressed either as a fixed text string or as a PROforma expression that is evaluated to yield a text string that can vary as the state of the process changes during its execution. The latter option can be used to create descriptions of arguments that vary according to the truth or falsity of the logical conditions associated with the argument.

### System design

#### Logical adequacy and heuristic power

In the system we consider here, PROforma process descriptions are considered as part of a larger software system. Hence some of the knowledge that the system contains is encoded in PROforma, whereas other parts are encoded in other components of the system, or held by human actors. This raises the question of what knowledge should be represented within a CIG, and what is more appropriately encoded outside of the CIG. For instance, in order to determine appropriate treatment of a patient enrolled in the study, it is necessary to estimate his or her risk of developing cardiovascular disease over a specified period. These risks are calculated using various measurements (e.g cholesterol levels) made at the beginning of the study. Therefore we must decide whether the PROforma process description should take, as input, the risk level itself, or the readings from which the risk level is calculated. If we adopted the latter option then the mathematical process by which one gets from the readings to the risk levels would be encoded within the PROforma process description and available for inspection by domain experts reading that process description. However we felt that setting out the calculation in this way would not be sensible, since it is part of a distinct and separate problem from that of deciding how to treat the patients enrolled in the study. Hence it seems more appropriate to calculate risk levels using another software component. Making these decisions, as part of the process of designing a software tool, is however a matter of judgement and we recognise that although the decision was not justified on the basis of the ease with which the assessment could be done in PROforma, the logical adequacy and heuristic support were factors in the decision since PROforma is designed to model processes and decisions, not inferences based on statistical models.

### System implementation

#### Logical adequacy

The implementation revealed two weaknesses in PROforma's logical adequacy.

• support for abstraction and information hiding

The PROforma Engine Interface permits inspection of all the data items and tasks in a PROforma Process Description, as well as all the values of all of their properties. It is however frequently useful to conceal some aspects of a Process Description and reveal others in order to distinguish between the essential logic of a process and the information that is required by some particular implementation of that logic. At present this distinction cannot easily be represented in PROforma.

• support for the definition of classes of tasks

The language allows the designer of a process to attach parameters to tasks in order to describe properties of those tasks that are important to the process but which are not built in to the PROforma language. This is important when Process Descriptions are embedded in a larger software system because it allows the other components of the system to query the process in a structured manner by reading the values of these parameters. However it is frequently the case that a Process Description will contain several tasks that have the same parameters and can therefore be regarded as forming a class. For instance tasks that involve altering a patent's medication might be grouped together into a class whose common properties are used to express the modifications needed. PROforma provides some facilities for the definition of task classes, but the expressive power of these facilities is limited (for instance they do not allow task classes to be grouped into hierarchies) and they are rather awkward to use.

#### Notational convenience

As noted above we used activity diagrams in the knowledge acquisition phase and translated these to PROforma in the implementation phase. When performing the translation we became aware of a limitation of PROforma. A transition between two states in a UML Activity Diagram can be given a guard condition and will only occur if that condition is satisfied. In PROforma transitions between tasks can be constrained using scheduling constraints, however it is not possible to attach a guard condition to a scheduling constraint. Instead preconditions are attached to the tasks themselves. It is possible to translate guarded transitions into PROforma by "migrating" the guard transition so that it becomes a precondition on a task, however this translation is not always straightforward because a precondition is always evaluated before a task commences whereas a guard is only evaluated when a state is entered via a particular transition.

#### Explanatory support

The translation from Activity Diagrams also made it harder to provide usable explanations because the Decisions, Arguments and Candidates in the PROforma process description used did not map directly on to the activity states and choice points identified in the activity diagrams created during analysis.

## Discussion

In this section we consider the most significant points indicated above under the four headings of logical adequacy, heuristic power, notational convenience, and explanation support and comment on the value of evaluation.

### Logical adequacy

In general we feel that the PROforma meets most of the demands of this application for logical adequacy in all three phases. The most serious shortcomings emerged in implementation and concern the lack of support for information hiding and data abstraction.

Consider the top-level plan shown in figure 3. It would be possible to modify this plan and yet argue that the essential logic of the process had not changed. For instance the three actions of the form "Return in *N *weeks" could be replaced by a single action containing a parameter that varied so as to express the value of *N*. Similarly the three decisions in the plan could be combined into a single decision with more complex logic. Ideally the Process Description would contain some sort of guarantee that such changes could not "break" any component of the system that accessed the process description. In order to do this it would be necessary to prevent "inessential" details of the process being revealed to other components of the system so as to provide a guarantee that these details could not affect their behaviour. At present there is no way of incorporating this kind of information hiding into a process description. The significance of this weakness becomes apparent when a complete system is created because the knowledge encoded in a process description often needs to be refined and one needs to know whether consequent changes will affect other components of the system.

### Heuristic power

Heuristic power describes the ability to draw inferences and solve problems within the intended application domains of the language. In order to decide whether requirements for heuristic power are met we must first decide what problems we expect to be able to solve and what type of inferences we expect to be able to draw. Such decisions affect the scope of guidelines and resulted in some of the most interesting reflections to emerge in the course of the study. One of the aims of the PROforma language is to describe the logic of processes in a way that allows the computerised form to be examined and validated by domain experts. Furthermore a PROforma Process Description is generally associated with a particular process and its authors envisage it being studied by a particular group of domain experts. Thus, when deciding whether to include knowledge within a Process Description, it is useful to ask whether those particular experts are in a position to validate it and, if so, whether it is sensible for it to be validated as part of a description of that particular process, or whether it should be decoupled and examined separately. The calculations used to derive estimates of cardiovascular risk are routinely used by expert cardiologists but their contents would have to be validated by a statistician or an epidemiologist.

Ethical, legal, and organisational considerations may also come into play when deciding whether to incorporate knowledge into a CIG. For instance stakeholders in a system may wish to consider who would be held responsible for any errors in advice provided, or the practicality of refining process descriptions if the knowledge involved was subject to frequent change. These factors had an impact on the advice provided by our system to GPs.

Also we observe that the PROforma expression language is not Turing complete. That is to say that there are calculations that could be performed by a Turing Machine (or expressed in a language such as Java or Pascal) but which cannot be expressed in the PROforma expression language. This is because the PROforma expression language does not provide any way of expressing functions that involve recursion or iteration (i.e. functions that would be expressed using a "do. .while" construct). It is possible to express such functions by attaching an expression to a task and then specifying that the task itself be executed in an iterative manner. However the use of such a mechanism to evaluate functions is clumsy, and obscures the logic of the guideline.

### Notational convenience

The most significant weakness was in the lack of a complete graphical notation for the language. This weakness is illustrated by the top-level plan set out in figure 3. By examining this figure we can see that there are, for example, circumstances under which a patient would be advised to return in two weeks time. However there is no standard way of indicating graphically what these circumstances are. The circumstances in question are encoded in the PROforma text of the process description and can be made visible through the use of appropriate software tools, but if one wants to convey information to a domain expert it is often much more convenient to draw a picture than to present him or her with a software tool, or with pages of text in an unfamiliar language.

By contrast the UML [26] has a complete graphical representation in the sense that every UML concept can be represented in a standard way in a UML diagram. This was one of the main reasons why UML activity diagrams were used during the knowledge acquisition phase of the project. In principle PROforma has many advantages over UML. For instance it can represent concepts such as arguments and candidates. Unfortunately there is no convention for representing them graphically and consequently it is hard to make use of these advantages.

Figure 5 represents the top-level plan as a UML Activity Diagram. The activity states of the form 'Return in *N *weeks' in the diagram correspond to the three actions of that form in the PROforma top level plan, as does the state 'Urgent Referral'. The state 'Flap Assessment' is nested (that is to say it contains a set of sub-states that are not shown in the diagram) and corresponds to the plan of the same name in the PROforma version. The decisions in the PROforma version are represented by choice points in the UML diagram, although there is not a one-to-one correspondence between decisions and choice points. An important advantage of the UML diagram is that there is a convention for expressing the conditions that have to be satisfied in order for a transition to occur between one state and another. They are represented as the 'guards', i.e. the text enclosed in square brackets on the arcs that connect states. In order to represent the same information in PROforma we would need to be able to represent the Candidates and Arguments of Decisions and the preconditions of Tasks in some graphical manner. It should be pointed out that the semantics of PROforma are better defined than those of UML diagrams. The relative imprecision of UML semantics did not prove a great drawback in this particular project. However had the logic of the system been more complex, we might have had to reconsider the use of UML as a modelling language.

Hederman et al. [27] have investigated the use of UML activity diagrams for the modelling clinical guidelines and have described a mapping between these diagrams and representations in GLIF.

### Explanatory support

We found the presentation of information in terms of the Arguments for favoured Candidates of Decisions was, broadly, a successful approach for the provision of explanations. However we experienced some difficulty in translating explanatory information from the UML Activity Diagrams to PROforma.

### Methodology

This paper uses a case study to investigate the PROforma language. It is perhaps worthwhile to compare and contrast our approach to that of the previous mentioned paper by Peleg et al. [21], who also used a case study to reveal the characteristics of Task Network Languages.

The most obvious difference between this paper and that of Peleg et al. is that we are only studying one language, PROforma, whereas Peleg et al. compared six languages. However this study embeds the PROforma process definitions in a real, distributed, system to be used in a clinical study, whereas contributors to the Peleg paper created Process Definitions, or guidelines, that were not used in this way and consequently did not have to be embedded in a larger software system. It is hoped that the more realistic demands placed on PROforma by the system studied in this paper will reveal strengths and weaknesses of the PROforma language that would not be apparent in a more theoretical study.

In particular we consider that strengths and weaknesses of the PROforma language are revealed by: the need to acquire the knowledge necessary to implement the system and to model this knowledge in a ways that can be both understood by domain experts and also implemented in a distributed computer system; the need to test and validate the knowledge so acquired; and the need to provide interfaces by which other components in a distributed system can access the state of the clinical processes that are modelled.

For instance the lack of graphical standards for PROforma emerges as an important issue when modelling knowledge acquired from experts because, at this stage of a project, it is necessary to be able to represent knowledge as diagrams on paper that can be read without the use of software tools. The need to hide information and create abstractions emerges when process descriptions are incorporated into larger systems because they provide a means of decoupling the process description from other components of the system and of limiting the changes to the system that are necessary when the process description is modified due to knowledge refinement.

It should be pointed out that the methodology used in this paper cannot be as systematic or as rigorous as that which would be expected when reporting, for example, a controlled clinical study. The criteria used for evaluation, such as notational convenience, have an unavoidable subjective element to them, and cannot be precisely measured.

## Conclusion

We have implemented a guideline-based decision support tool as a part of a research trial exploring the role community pharmacists could play in the management of hypertension. The development of the system involved a knowledge engineering exercise in which PROforma, a process description language designed specifically for capturing clinical guidelines, was used to represent the trial protocol. Although PROforma was used for knowledge representation, UML diagrams were used in the knowledge acquisition process.

The PROforma representation was successfully employed as part of a web-based tool in which the PROforma engine was integrated with other software elements, notably a secure database of patient information.

This case study reveals a number of important lessons about guideline representation languages such as PROforma. These include a number of limitations in logical adequacy, but no difficulty with heuristic power. Most importantly, the experience accumulated during this study has highlighted the importance of structure preserving design in the construction of systems that make use of computer interpretable guidelines. A consequence of this is that great importance is attached to the notational convenience of the languages in which these guidelines are expressed. It is particularly important that the languages should allow the construction of complete and unambiguous graphical representations of guidelines.

## Competing interests

David Sutton was one of the principal designers of the Tallis system and is occasionally employed as a consultant by Cancer Research UK.

## Authors' contributions

DS implemented the software used in this study and is the primary author of this paper. PT worked extensively on the background section to the paper and helped to set the other sections in the context of existing research, as well as clarifying and expanding them. KE and PT instigated and designed the hypertension study for which the software was developed. All authors read and approved the final manuscript.

## Pre-publication history

The pre-publication history for this paper can be accessed here:


